# Three endoplasmic reticulum-associated fatty acyl-coenzyme a reductases were involved in the production of primary alcohols in hexaploid wheat (*Triticum aestivum* L.)

**DOI:** 10.1186/s12870-018-1256-y

**Published:** 2018-03-05

**Authors:** Guaiqiang Chai, Chunlian Li, Feng Xu, Yang Li, Xue Shi, Yong Wang, Zhonghua Wang

**Affiliations:** 1 State Key Laboratory of Crop Stress Biology for Arid Areas, Northwest A&F University, Yangling, Shaanxi 712100 China; 2 College of Agronomy, Northwest A&F University, Yangling, Shaanxi 712100 China

**Keywords:** Hexaploid wheat, Cuticular wax, Primary alcohol, Endoplasmic reticulum, Fatty acyl-coenzyme A reductase

## Abstract

**Background:**

The cuticle covers the surface of the polysaccharide cell wall of leaf epidermal cells and forms an essential diffusion barrier between the plant and the environment. The cuticle is composed of cutin and wax. Cuticular wax plays an important role in the survival of plants by serving as the interface between plants and their biotic and abiotic environments, especially restricting nonstomatal water loss. Leaf cuticular waxes of hexaploid wheat at the seedling stage mainly consist of primary alcohols, aldehydes, fatty acids, alkane and esters. Primary alcohols account for more than 80% of the total wax load. Therefore, we cloned several genes encoding fatty acyl-coenzyme A reductases from wheat and analyzed their function in yeast and plants. We propose the potential use of these genes in wheat genetic breeding.

**Results:**

We reported the cloning and characterization of three *TaFARs,* namely *TaFAR6*, *TaFAR7* and *TaFAR8*, encoding fatty acyl-coenzyme A reductases (FAR) in wheat leaf cuticle. Expression analysis revealed that *TaFAR6*, *TaFAR7* and *TaFAR8* were expressed at the higher levels in the seedling leaf blades, and were expressed moderately or weakly in stamen, glumes, peduncle, flag leaf blade, sheath, spike, and pistil. The heterologous expression of three *TaFARs* in yeast (*Saccharomyces cerevisiae*) led to the production of C24:0 and C26:0 primary alcohols. Transgenic expression of the three *TaFARs* in tomato (*Solanum lycopersicum*) and rice (*Oryza sativa*) led to increased accumulation of C24:0–C30:0 primary alcohols. Transient expression of GFP protein-tagged TaFARs revealed that the three TaFAR proteins were localized to the endoplasmic reticulum (ER), the site of wax biosynthesis. The three *TaFAR* genes were transcriptionally induced by drought, cold, heat, powdery mildew (*Blumeria graminis*) infection, abscisic acid (ABA) and methyl jasmonate (MeJa) treatments.

**Conclusions:**

These results indicated that wheat TaFAR6, TaFAR7 and TaFAR8 are involved in biosynthesis of very-long-chain primary alcohols in hexaploid wheat and in response to multiple environmental stresses.

**Electronic supplementary material:**

The online version of this article (10.1186/s12870-018-1256-y) contains supplementary material, which is available to authorized users.

## Background

Plants are affected by poor environmental conditions, which interrupt normal growth and productivity. Drought is one of the most important abiotic stresses which causes osmotic stress in plants and profoundly affects crop production worldwide each year [1]. Plants respond to abiotic stresses by changing the varieties of cellular processes [2–4], and wax secretion from the plant epidermal cells to cuticle represents one of these significant changes. The cuticle, which covers most of the surfaces of terrestrial plants, mainly consists of cutin and cuticular wax. Insoluble cutin is the primarily structural component of the cuticle and is a polymer consisting of ω- and midchain hydroxy and epoxy C_16_ and C_18_ fatty acids connected by ester bonds and glycerol bridges. Insoluble cutin constitutes 40–80% of the cuticle mass [5–7]. Cuticular waxes are complex mixtures of lipids that are composed of very-long-chain aliphatic molecules and theirs derivative, including primary and secondary alcohols, aldehydes, alkanes, ketones, esters, triterpenoids, sterols and flavonoids [8, 9]. The cuticular wax plays an important role in the survival of plants, serving as the interface between plants and their biotic and abiotic environments. The primary physiological function of cuticular wax is to seal the tissue against a relatively dry atmosphere, preventing desiccation by minimizing nonstomatal water loss [10, 11]. In addition, cuticular wax protects plants from UV radiation [12–15]. Cuticular wax also protects against plant pathogens and insects [16].

The biosynthesis of cuticular wax begins with C_16_ or C_18_ de novo fatty acid synthesis on the outer membrane of epidermal cells in the plastid. Then, using C_16_ and C_18_ acyl-CoA and malonyl-CoA as substrates, the fatty acid elongase (FAE) complex performs a reiterative cycle of four reactions catalyzed by a β-ketoacyl-CoA synthase (KCS), a β-ketoacyl-CoA reductase (KCR), a β-hydroxyacyl-CoA dehydratase (HCD), and an enoyl-CoA reductase (ECR) to synthesize saturated very-long-chain fatty acids (VLCFAs) [17]. These VLCFAs are then further modified into various wax molecules through two major pathways: the acyl-reduction and the decarbonylation pathways [18]. Alcohols and esters are products in the acyl-reduction pathway, whereas alkanes, aldehydes, secondary alcohols and ketones are components of the decarbonylation pathway.

Bread wheat (*Triticum aestivum L.*2n = 42; AABBDD) is a major food crop worldwide. At seedling stage, leaf cuticular waxes of hexaploid wheat mainly consist of primary alcohols, aldehydes, fatty acids, alkane and esters. Primary alcohols accounted for more than 80% of the total wax load, and C_28_ primary alcohol is a major component of primary alcohols [19–21]. Thus, the acyl-reduction pathway mainly participates in this process. Fatty-acyl coenzyme A reductase (FAR) is a crucial enzyme involved in the biosynthesis of very long chain fatty alcohols [22]. Several FARs from plants have been characterized, such as *jojoba*, *Arabidopsis thaliana* and wheat. In jojoba, the FAR could catalyze VLCFA precursors to fatty alcohols [23]. Eight genes encoding putative FARs have been identified in Arabidopsis, and the biological functions of five of the corresponding proteins have been described. AtFAR2 produces primary alcohols that are incorporated into sporopollenin of the pollen exine layer [24, 25], while *CER4* (*AtFAR3*) generates C24:0–C30:0 primary alcohols present in the cuticular wax of aerial tissues [22]. Furthermore, three *FARs* (*AtFAR1*, *AtFAR4* and *AtFAR5*) catalyze the formation of the 18:0, 20:0 and 22:0 fatty alcohols found in suberin polyester and root waxes [26]. Several FARs have been identified in gramineous model crops. In rice, DPW and OsFAR6/1/4 participated in primary alcohol biosynthesis [27, 28]. *Brachypodium distachyon* BdFARs have also proven to possess FARs activity [29]. In hexaploid wheat, *TaFAR1*, *TaFAR5*, *TaTAA1a*, *TaFAR2*, *TaFAR3* and *TaFAR4* have been identified. These genes all encoded FARs and participated in biosynthesis of fatty alcohols. *TaFAR1* and *TaFAR5* could produce C26:0, C28:0 and C30:0 fatty alcohols when expressing in tomato leaves [30, 31]. *TaTAA1a*, an anther-special gene, encodes a FAR related to pollen fertility [32]. *TaFAR2*, *TaFAR3* and *TaFAR4* catalyze the biosynthesis of C18:0, C28:0 and C24:0 fatty alcohols in yeast, respectively [33]. However, only a few genes produce C_28_ primary alcohol; therefore, other *FAR* genes may be involved in biosynthesis of fatty alcohols in wheat.

To further elucidate the molecular mechanisms of the other *FARs* from wheat, the aims of our study were as follows: (1) to clone and characterization three *TaFARs* from hexaploid wheat; (2) to identify whether these FARs to encode a functional FAR enzyme that catalyzes the production of very-long-chain primary alcohols in yeast; (3) to identify whether these *TaFARs* are responsible for fatty alcohol formation in transgenic tomato (*Solanum lycopersicum* cv. MicroTom) and rice (*Oryza sativa* L.); (4) to determine their subcellular location and expression patterns.

## Methods

### Plant materials and growth conditions

Hexaploid wheat Xinong979, tomato variety Micro-Tom (*Solanum lycopersicum* L.) and rice variety Zhonghua11 (*Oryza sativa* L. spp. *japonica*) were kindly provided by Crop Molecular Biology and Breeding laboratory, College of Agronomy, Northwest A&F University, China. Among these materials, Xinong 979, a major winter wheat cultivar planted in the southern area of the Huang-huai wheat region in China, was used throughout the experiments for gene cloning and expression. Tomato variety Micro-Tom and rice variety Zhonghua11 were used for genetic transformation. For gene expression analysis, Xinong979 was grown in field during the 2014–2015 wheat-growing seasons. The young leaves were collected at seedling stage, and the root, peduncle, flag leaves, sheath, glume, spike, anther and pistil were collected at flowering stage. For abiotic stress analysis, the Xinong979 seedlings were grown in a greenhouse for 4–5 weeks and then subjected to various stresses. All abiotic stress treatments were conducted as described previously [31].

### Cuticular wax extraction, chemical components and scanning electron microscopy (SEM) analysis

The tomato leaves and fruits, rice leaves and sheaths were harvested and immersed in chloroform for 50 s in a fume cupboard. After extraction, 20 μg *n*-tetracosane was added to each sample as an internal standard. The samples were transferred to a gas chromatograph (GC) autosampler vial and dried under a stream of nitrogen gas. For GC analysis, the wax samples were derivatized with 45 μL pyridine (Sigma) and 45 μL N,O-bis (trimethylsilyl)-trifluoroacetamide (BSTFA) (Sigma) for 1 h at 70 °C. Then, the sample was dried under nitrogen gas before being redissolved in 500 μL of chloroform. The GC equipped with a mass spectrometric detector was used for qualitative analysis, and flame ionization detector (FID) was used for quantitative analysis.

For SEM analysis, the cuticular tissues were harvested and dried under 50 °C for 1 week before 5–10 mm completely dried pieces were attached with double adhesive tape to the aluminum stubs and sputter-coated with gold particles. The samples were observed using a SEM (Hitachi S4800) at an accelerating voltage of 10 kV and a working distance of 12 mm.

### RNA isolation, cDNA synthesis and quantitative real-time PCR (qRT-PCR) analysis

Total RNA from hexaploid wheat samples was extracted using TRIzol™ Reagent (TaKaRa Biotechnology, Dalian Co., Ltd., China) according to the manufacturer’s instructions. The potential contaminating DNA in total RNA was digested with *DNase I*. First-strand cDNA was synthesized using the GoScript Reverse Transcription System (TaKaRa). Oligo(dT)18 was used as a primer. The reverse transcription reaction was incubated at 42 °C for 1 h followed by 70 °C for 15 min in a total volume of 20 μL. After a 1:20 dilution, 0.5 μL of the synthesized cDNA was used for RT-PCR or qRT-PCR. The primers for PCR were designed based on the cDNA sequence (Additional file 1: Table S1), and qRT-PCR was conducted as described previously [34]. To standardize the data, the wheat *ACTIN* gene (*TaACTIN* GenBank accession number AB181991) was used as an internal reference for the qRT-PCR analysis. Quantification of gene expression was performed using a CFX96 Real-Time PCR System (Bio-Rad). Dissociation curves were generated for each reaction to ensure specific amplification. All reactions, including the negative control, were performed three times. The threshold values (*CT*) generated from the CFX96 Real-Time software tool were employed to quantify the relative gene expression using the comparative threshold (2^−ΔΔ*CT*^) method [35]. Three independent biological replicates were also performed for each experiment.

### Cloning and comparative analyses of *TaFAR6*, *TaFAR7* and *TaFAR8*

The protein sequence of CER4 (NC_003075) reported by Rowland [22] was aligned in National Center of Biotechnology Information (NCBI) via tBlastn. The protein shared high homologous identity with the uncharacterized proteins derived from wheat, but the function of the proteins was unknown. Thus, three pairs of special primers (Additional file 1: Table S1) were designed for amplification of *TaFARs*. The PCR products were purified, cloned into the pMD18-T vector (TaKaRa) and sequenced.

The cDNA sequences were analyzed using Lasergene7 (DNASTAR) and blast (https://blast.ncbi.nlm.nih.gov/Blast.cgi) to search the Non-Redundant (NR). Pfam (http://pfam.xfam.org/), InterProScan (http://www.ebi.ac.uk/interpro/search/sequence-search) and PROSITE Scan (https://prosite.expasy.org/scanprosite/) were used to predict conserved domains or motifs. Multiple sequence alignments were performed by ClustalW. Phylogenetic trees of FARs were constructed by MEGA7.0 software.

### Heterologous expression of *TaFARs* in yeast (*Saccharomyces cerevisiae*)

The coding sequences of *TaFARs* were amplified from cDNA of wheat cv. Xinong979 using a pair of primers labeled as pYES-TaFARs-7F and pYES-TaFARs-7R (Additional file 1: Table S1). The PCR fragment was cloned into the yeast expression vector pYES3. Then, the recombinant plasmid was transformed into *E. coli* DH5α and verified by sequencing. Subsequently, the two vectors pYES3-TaFARs and P416 were co-transformed into yeast mutant strain INVSc1 (MATα his3-Δ1 leu2 trp1–289 ura3–52), and the empty pYES3 vector was used as the negative control. Transgenic yeast cells were selected by growth on uracil and tryptophan deficient synthetic complete (SC-Ura-Trp) medium with 2% (*w*/*v*) glucose at 30 °C. Three individual colony were induced and lipids were extracted using the protocol previously reported [36].

### Overexpression of *TaFARs* in tomato and rice

To generate the overexpression vector for *TaFAR6*, *TaFAR7* and *TaFAR8*, the entire ORFs of three *FAR* genes were amplified using primer pair *TaFARs*-F/*TaFARs*-R and cloned into pCXSN, which was driven by the Cauliflower Mosaic Virus (CaMV) 35S promoter. The overexpression vectors were introduced into *Agrobacterium tumefaciens* strain GV3101 and EHA105. The resulting strains were used to transform the tomato cv. MicroTom and japonica cv. Zhonghua 11 as previously reported [37, 38]. All transgenic plants were planted in greenhouse. The phenotypes and cuticular wax analysis of the transgenic plants were examined in the T_1_ generation.

### Subcellular localization of TaFARs

The coding sequences of *TaFAR6*, *TaFAR7* and *TaFAR8* without the termination codons were cloned and recombined into the N-terminus of sequences encoding specific fluorescent proteins under the control of the CaMV35S promoter. Briefly, the endoplasmic reticulum (ER) marker mCherry-HDEL and the fusion construct TaFARs-GFP were used in co-localiztion assays. Vectors were transferred into epidermal cell of tobacco using the bombardment-mediated method [39, 40] and incubated for 24 h in the dark at room temperature to allow transient expression. The transformed epidermal cells were observed using a confocal laser scanning microscope (LSM700; Zeiss, Germany) at the following excitation wavelengths: mCherry at 564 nm and GFP at 488 nm.

## Results

### Identification of *FAR* genes from hexaploid wheat and phylogenetic analysis

The previous studies indicated that the primary alcohols were the dominant components of cuticular wax on wheat leaves at the seedling stage [31, 41]. In order to clone the genes related to fatty alcohol biosynthesis, a Blast search of the wheat GenBank database was performed using the amino acid sequence of arabidopsis *CER4* (GenBank accession No. NP_567936) which encodes an alcohol-forming FAR. Finally, ten candidate sequences were identified, and all exhibit high similarity to *CER4* over their entire length. Six of these candidate sequences, *TaFAR1*, *TaTAA1a*, *TaTAA1b*, *TaTAA1c*, *TaFAR2* and *TaFAR3,* have been reported, but the biological function of the other four genes have not been identified. The most highly represented genes, *TaFAR6*, *TaFAR7* and *TaFAR8* (GenBank accession No.MF804951, MF817443, MF817444), were selected for further research in this study. Subsequently, the cDNA fragments encoding *TaFAR6*, *TaFAR7* and *TaFAR8* were isolated from leaf blades of hexaploid wheat cv. Xinong979. Sequence analysis revealed that *TaFAR6*, *TaFAR7* and *TaFAR8* contain open reading frames of 1497, 1503 and 1479 bp that encode encoding 499, 501 and 493 amino-acid residues with the predicted protein molecular weight of 56.9, 57.4 and 55.6 kD, respectively.

**Fig. 1:**
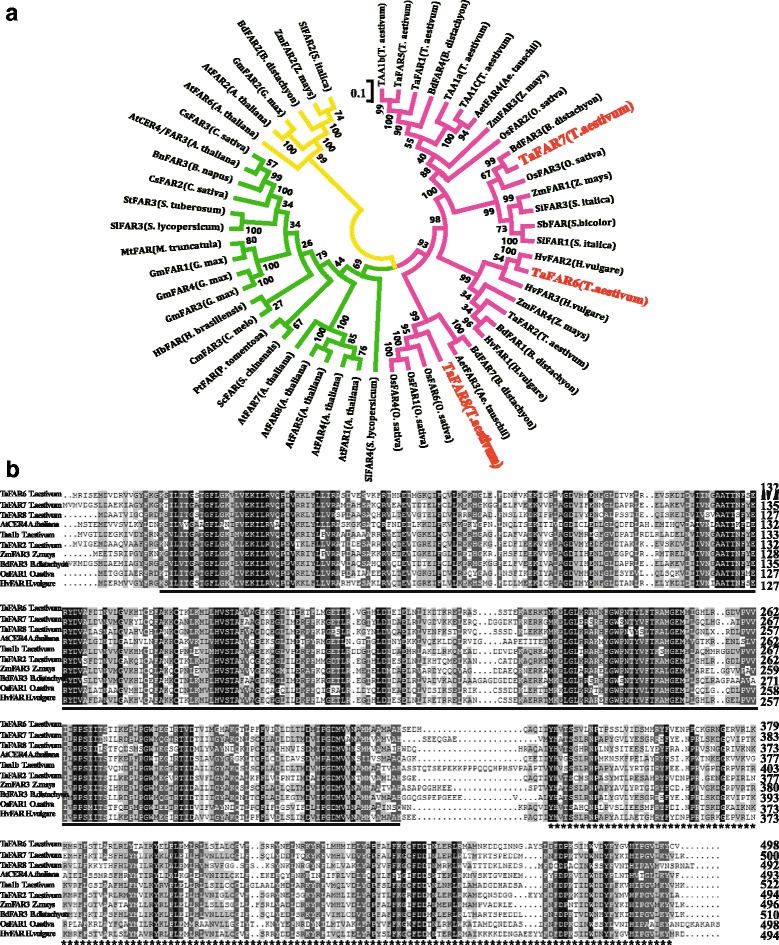
Phylogenetic analysis and multiple sequence alignment of TaFARs. **a** Phylogenetic tree of alcohol-forming FARs from wheat cv. Xinong979 and other plant species, including monocotyledons and dicotyledons. The phylogenetic analysis was conducted by MEGA7.0 software using the Neighbor-Joining method. **b** Multiple alignment of wheat TaFAR6, TaFAR7 and TaFAR8 with five related proteins using the DNAMAN software. NAD_binding_4 and sterile domains are indicated by black lines and stars under the sequences, respectively

The functional domain for TaFARs was predicted using online CDD software from the NCBI (https://www.ncbi.nlm.nih.gov/Structure/cdd/wrpsb.cgi). The results showed that three TaFARs proteins contain a NADB binding domain at the N-terminal linked with a FAR_C domain at the C-terminal. These two domains were consistent with FARs from other animal and plant species, such as *Euglena* EgFAR [42] and wheat TaFAR1 and TaFAR5 [30, 31]. Multiple sequence alignment revealed that TaFAR6, TaFAR7 and TaFAR8 exhibited 50–55% identity with five different plant species homologues: AtCER4, TAA1b, ZmFAR3, BdFAR3 and OsFAR1 (Fig. 1b). Phylogenetic analysis of 55 proteins with similarities to TaFAR6, TaFAR7 and TaFAR8 showed that all proteins can be grouped into three clades. TaFAR6, TaFAR7 and TaFAR8 were grouped into the first clade, which was derived from the monocot (Fig. 1a). TaFAR8 had the highest homology with three rice proteins OsFAR6, OsFAR1 and OsFAR4. TaFAR6 and TaFAR7 were the most similar to HvFAR2 and BdFAR3, respectively. These results suggest that the TaFAR6, TaFAR7 and TaFAR8 proteins possess FAR activity associated with primary alcohol biosynthesis in wheat.

### Analysis of *TaFARs* gene expression

**Fig. 2:**
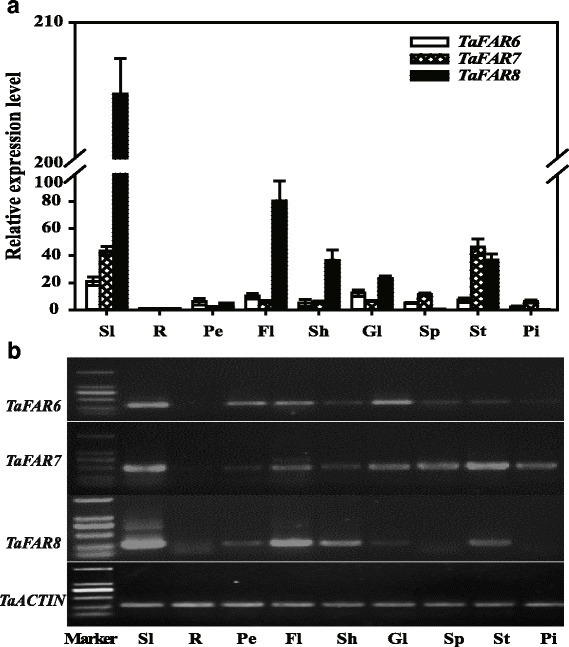
Expression of *TaFARs* in different tissues of hexaploid wheat cv. Xinong979. **a** qRT-PCR analysis of *TaFARs* expression in various organs of wheat. Sl, seedling leaf blade; R, root; Pe, peduncle; Fl, flag leaf blade; Sh, sheath; Gl, glume; Sp, spike; St, stamen; Pi, pistil. Wheat *TaACTIN* was used as reference. Error bars indicate the SE of three independent replicated samples. **b** Semi-quantitative analysis of *TaFARs* expression in various organs of wheat

RT-PCR and qRT-PCR were performed to investigate the transcription profile of the three *TaFARs* in various whole tissues. The samples were derived from 2-month-old seedling leaves and various organs at the flowering stage. The *TaFARs* were expressed in all tissues except root tissue. *TaFAR6* was highly expressed in seedling leaf blades and glumes, modestly expressed in peduncle and flag leaf blade, and weakly expressed in sheath, spike, stamen and pistil. *TaFAR7* mainly showed a relatively high expression level in seedling leaf blades, spike and stamen compared with other tissue organs. *TaFAR8* exhibited increased expression in the seedling leaf blade and was highly expressed in flag leaf blade, sheath and stamen (Fig. 2a, b). Furthermore, we generated a bacterial expression vector by inserting the *TaFAR* coding region into the pET28a vector (Novagen) and introducing this construct into *E. coli* BL21 (DE3). Consistent with the predicted size, the molecular mass of TaFAR6, TaFAR7 and TaFAR8 proteins were estimated to be 56.8, 57.4 and 55.6 kD, respectively, by SDS-PAGE analysis (Additional file 2: Figure S2).

**Fig. 3:**
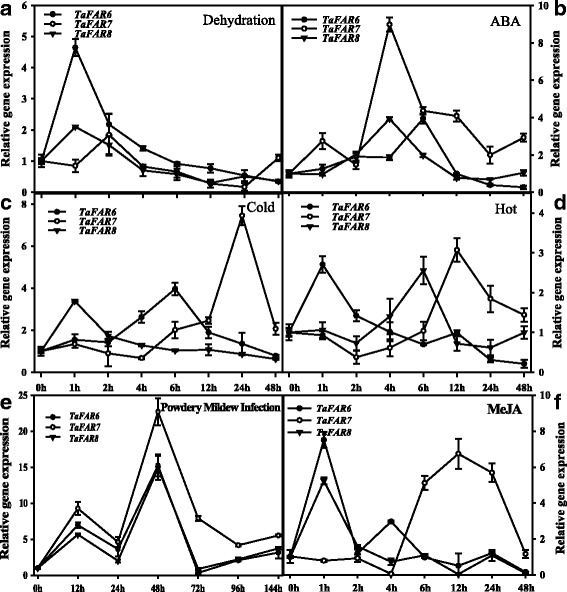
Expression analysis of *TaFAR6*, *TaFAR7* and *TaFAR8* under various stresses conditions. Wheat cv. Xinong979 seedlings approximately four-week-old were exposed to stress treatments. **a** Dehydration treatments. **b** ABA treatment, 100 μM ABA. **c** Cold treatment, 4 °C. **d** Heat treatment, 42 °C incubator. **e** Powdery mildew infection. **f** MeJA treatment, MeJA 100 μM

Previous studies indicated that the plant cuticular wax can be regulated by a range of abiotic stresses [43–45]. Transcript profiling from leaves of pot-grown wheat plants showed that *TaFAR6*, *TaFAR7* and *TaFAR8* genes were responsive to dehydration, cold, heat, ABA hormone, powdery mildew infection and MeJa treatment (Fig. 3). When the seedlings were exposed to dehydration stress, *TaFAR7* transcript levels peaked at 2 h, whereas those of *TaFAR6* and *TaFAR8* peaked at 1 h (Fig. 3a). After ABA treatment, *TaFAR7* and *TaFAR8* showed the highest expression levels at 4 h, while *TaFAR6* peaked at 6 h (Fig. 3b). Expression of *TaFAR7* slowly increased within 12 h after initiation of cold treatment, and its transcript level peaked at 24 h. However, the accumulation of *TaFAR6* and *TaFAR8* mRNA rapidly increased, and their expression levels peaked at 1 h and 6 h, respectively (Fig. 3c). Under heat stress condition, three *TaFARs* were induced, and *TaFAR6* mRNA reached its highest level at 1 h. In contrast, the highest transcript levels for *TaFAR7* and *TaFAR8* occurred at 12 h and 6 h, respectively (Fig. 3d). *TaFAR6*, *TaFAR7* and *TaFAR8* expression increased gradually within 24 h after powdery mildew infection and then rapidly declined over 24 h (Fig. 3e). Finally, we also exposed wheat seedlings to MeJA, which acts as an essential signal involved in defense/stress pathway in monocots [46, 47]. *TaFAR6*, *TaFAR7* and *TaFAR8* expression was strongly induced by MeJA and peaked at 1 h. Compared with *TaFAR6* and *TaFAR8*, *TaFAR7* transcript levels dramatically increased until 6 h after MeJA treatment and peaked at 12 h (Fig. 3f). The results suggested that *TaFAR6*, *TaFAR7* and *TaFAR8* were induced by multiple environmental stresses and participated in the ABA and MeJA-dependent stress signal pathway.

### *TaFAR* expression in yeast

**Fig. 4:**
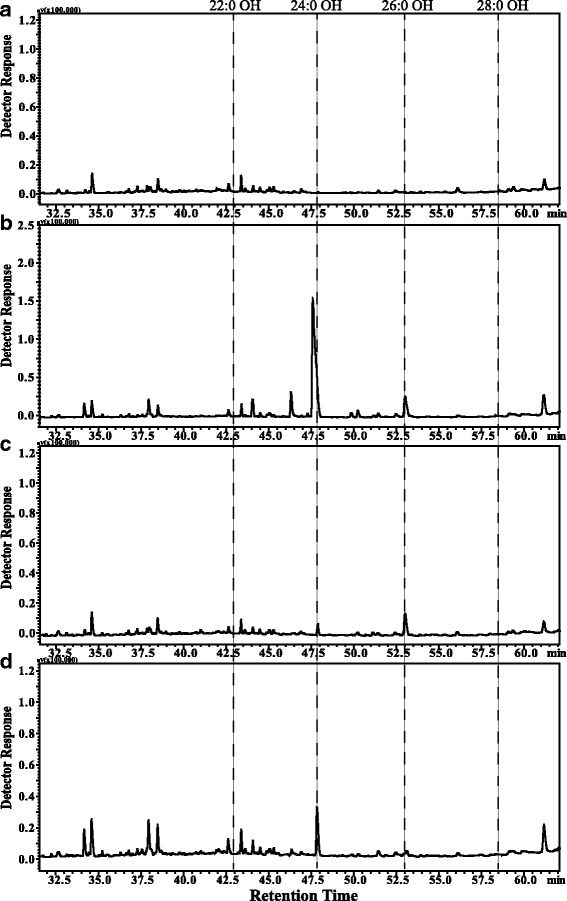
Heterologous expression of the TaFAR6, TaFAR7 and TaFAR8 in yeast. Yeast was transformed with empty vector control pYES3 (**a**) or vector harboring TaFAR6 (**b**), TaFAR7(**c**) and TaFAR8 (**d**). Transgenic yeast cell were grown in stringent medium lacking tryptophan and uracil. Lipids were extracted from yeast and analyzed by GC

In order to confirm the three *TaFARs* were alcohol-forming FARs involved in the production of very-long-chain primary alcohols, we expressed the ORFs of three *TaFAR* genes in the yeast under the control of GAL1 promoter. The recombinant plasmid pYES3-TaFARs and p416 were co-transformed into the yeast mutant strain INVSc1 (Invitrogen), and the corresponding empty vector was used as the negative control. Yeast cells were induced and lipids were extracted according to the previous reports [48]. GC-MS results showed that no alcohols were detected when the empty vector was expressed in yeast (Fig. 4a). Based on authentic standards and MS characteristics, C_24_ and C_26_ primary alcohols were identified when TaFAR6 was expressed in transgenic yeast (Fig. 4b). Interestingly, C_24_ and C_26_ primary alcohols were also identified when TaFAR7 and TaFAR8 were expressed in transgenic yeast, respectively (Fig. 4c, d; Additional file 3: Figure S3). These results demonstrated that these three TaFARs possessed FAR activity and catalyzed the biosynthesis of very-long-chain primary alcohols.

### The expression of three *TaFARs* in tomato results in primary alcohol accumulation

**Fig. 5:**
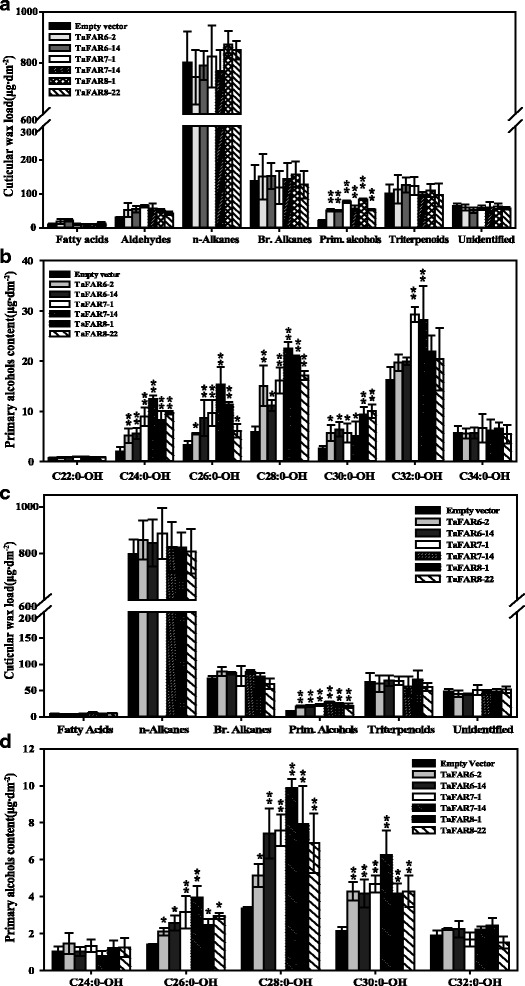
Cuticular wax analysis of tomato fruits and leaves overexpressing *TaFAR6*, *TaFAR7* and *TaFAR8*. **a** Total fruit wax coverage in different compound classes. Data are expressed as μg per dm^2^. **b** Absolute amount (μg⋅dm^− 2^) of primary alcohols in fruits. **c** Total leaf wax coverage in different compound classes. **d** Absolute amount (μg⋅dm^− 2^) of primary alcohols in leaves. Values are means of *n* ≥ 4 biological replicates. Error bars = SD. Asterisks represent significant differences between *TaFAR* expression vector and empty vector control (t-test: * for *p* < 0.05; ** for *p* < 0.01)

In order to further confirm that the *TaFAR6*, *TaFAR7* and *TaFAR8* were FARs catalyzing the accumulation of very-long-chain primary alcohols, we constructed three overexpression vectors pCXSN-TaFARs controlled by the cauliflower mosaic virus (CaMV) 35S promoter (Additional file 4: Figure S4a), and these gene products were expressed in dicotyledon tomato and monocotyledon rice (Additional file 4: Figure S4b, e). The transgenic lines harboring empty vector were used as the negative control. No significant morphological differences were observed between transgenic lines and control lines (Additional file 4: Figure S4a). The cuticular wax composition of the red fruit and mature leaves from T_1_ generation transgenic lines were analyzed by GC-FID. As expected, the total primary alcohol content was dramatically increased in six transgenic lines compared with control lines, whereas the levels of fatty acid, aldehydes, *n*-alkanes, branched alkanes, and triterpenoids were only slightly affected in the transgenic lines (Fig. 5a, c).

In fruits, the absolute content of total primary alcohols increased from 20.62 μg/dm^2^ in the fruit cuticle of the control line to 52.15 μg/dm^2^ in transgenic line TaFAR6–2, 77.20 μg/dm^2^ in line TaFAR7–1 and 84.10 μg/dm^2^ in line TaFAR8–1 (Fig. 5a). Subsequently, the chain length of primary alcohols was further analyzed, and the results revealed that C_24_, C_26_, C_28_ and C_30_ primary alcohols were increased in transgenic lines expressing *TaFAR6* and *TaFAR8* (Fig. 5b). Similar chain length patterns for primary alcohols were also observed in TaFAR7 transgenic lines except that the content of C_32_ primary alcohol was also dramatically increased compared with the control line. In the transgenic line TaFAR6–2, the greatest increase in the absolute contents of C_24_, C_26_, C_28_ and C_30_ primary alcohols, which exhibited increases of 1.6-, 0.7-, 1.5- and 1.2-fold, respectively, compared with the control line. Similar results were noted in the transgenic line TaFAR7–1. The wax mixtures on ripe fruit showed significant increases in primary alcohols with a 3.5-fold increase in C_24_ primary alcohol, a 2.0-fold increase in C_26_ primary alcohol, a 1.7-fold increase in C_28_ primary alcohol, a 1.2-fold increase in C_30_ primary alcohol, and a 0.8-fold increase in C_32_ primary alcohol. Interestingly, the C_24_, C_26_, C_28_ and C_30_ primary alcohols were also found to be increased by 3.5-, 2.5-, 2.4- and 2.7-fold in the transgenic line TaFAR8–1, respectively (Fig. 5b).

In leaves, the wax load per unit of leave area of TaFAR6–2, TaFAR7–1 and TaFAR8–1 were 19.63, 22.75 and 23.51 μg/dm^2^, which is equivalent to 2-, 1.8-, 3-fold increase compared with the control lines, respectively (Fig. 5c). The chain length of primary alcohols was also further analyzed, and the results revealed that C_26_, C_28_ and C_30_ primary alcohols were dramatically increased in all transgenic lines (Fig. 5d).

In addition, in order to further determine whether the differences in the primary alcohol content in the transgenic lines influence the alteration of the crystal pattern, SEM revealed that substantially more cuticular wax crystals were deposited on the surface of the fruits epidermal cells in overexpression plants compared with CK plants (Additional file 5: Figure S5a–d). However, no evident crystal structure changes were observed on the surface of the epidermis of leaves (Additional file 5: Figure S5e–l). These primary alcohol changes provided further evidence that the three TaFARs are alcohol-forming FARs that are responsible for the very-long-chain alcohols formation in the epidermal cells of tomato fruits.

### Overexpression of three *TaFARs* altered the content of primary alcohols in rice

**Fig. 6:**
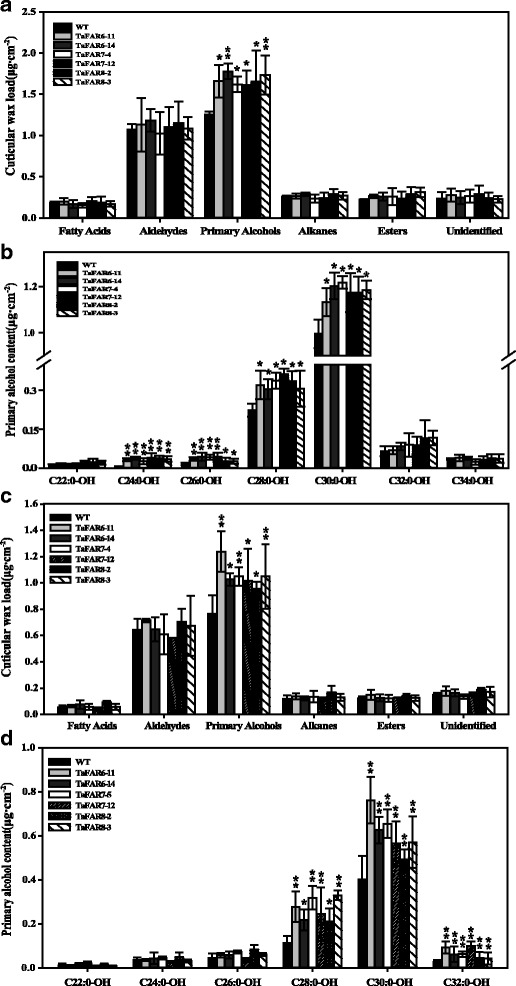
Cuticular wax analysis of rice leaf and sheath overexpressing *TaFAR6*, *TaFAR7* and *TaFAR8*. **a** Total leaf wax coverage in different compound classes. Data are expressed as μg per cm^2^. **b** Absolute amount (μg⋅cm^− 2^) of primary alcohols in leaves. **c** Total sheath wax coverage in different compound classes. **d** Absolute amount (μg⋅cm^− 2^) of primary alcohols in sheath. Values are means of *n* ≥ 4 biological replicates. Error bars = SD. Asterisks represent significant differences between *TaFAR* expression vector and empty vector control (Student’s test was performed: * for *P* < 0.05; ** for *P* < 0.01)

To further confirm that the *TaFARs* were involved in cuticular wax biosynthesis, we also expressed these three *TaFAR* genes in rice cv. Zhonghua11. No significant morphological differences were noted between transgenic lines and CK lines (Additional file 4: Figure S4c), and cuticular wax composition of flag leaves from T_1_ generation transgenic lines were analyzed by GC-FID. As anticipated, a significant difference was detected on leaves of transgenic plants (Fig. 6a). Total primary alcohols were significantly increased, and other wax components exhibited no obvious changes. Then, the chain length of primary alcohols was further analyzed. The results revealed that C_24_, C_26_, C_28_ and C_30_ primary alcohols were dramatically increased in transgenic lines of TaFAR6, TaFAR7 and TaFAR8, compared with the control line (Fig. 6b). In transgenic line TaFAR6–14, the greatest increases were noted in the absolute contents of C24:0-OH, C26:0-OH, C28:0-OH and C30:0-OH, which exhibited increases of 4.2-, 1.8-, 0.4- and 0.3-fold, respectively, compared with control line. Interestingly, similar results occurred in transgenic line TaFAR7–12 and TaFAR8–2.

Subsequently,the sheath wax of rice was also detected. Similar results were found, and the wax mixtures on the sheath showed significant increases in primary alcohols but not in other compounds classes (Fig. 6c). The most drastic effect was observed for the C_28_, C_30_ and C_32_ primary alcohols, all of which exhibited approximately 0.5- to 1-fold increase in independent transgenic lines compared with the control line (Fig. 6d). Finally, SEM was used for a detailed examination of the surfaces. In control lines, the adaxial and abaxial leaf blade surfaces were densely covered with platelet-type wax crystals including the unevenly distributed cuticular papillae. Transgenic rice lines overexpressing *TaFAR6*, *TaFAR7* and *TaFAR8* exhibited an obvious increase of wax crystals on both adaxial and abaxial sides of the leaf blade surface (Additional file 6: Figure S6a-h). Fortunately, there were also evident crystal structure changes on the surface of sheath epidermis (Additional file 6: Figure S6i-l). These results provide further confirmation that these three TaFARs were active FAR, likely accepting C_24_ to C_30_ fatty acyl-CoAs as substrates in rice.

### TaFARs located in the ER

**Fig. 7:**
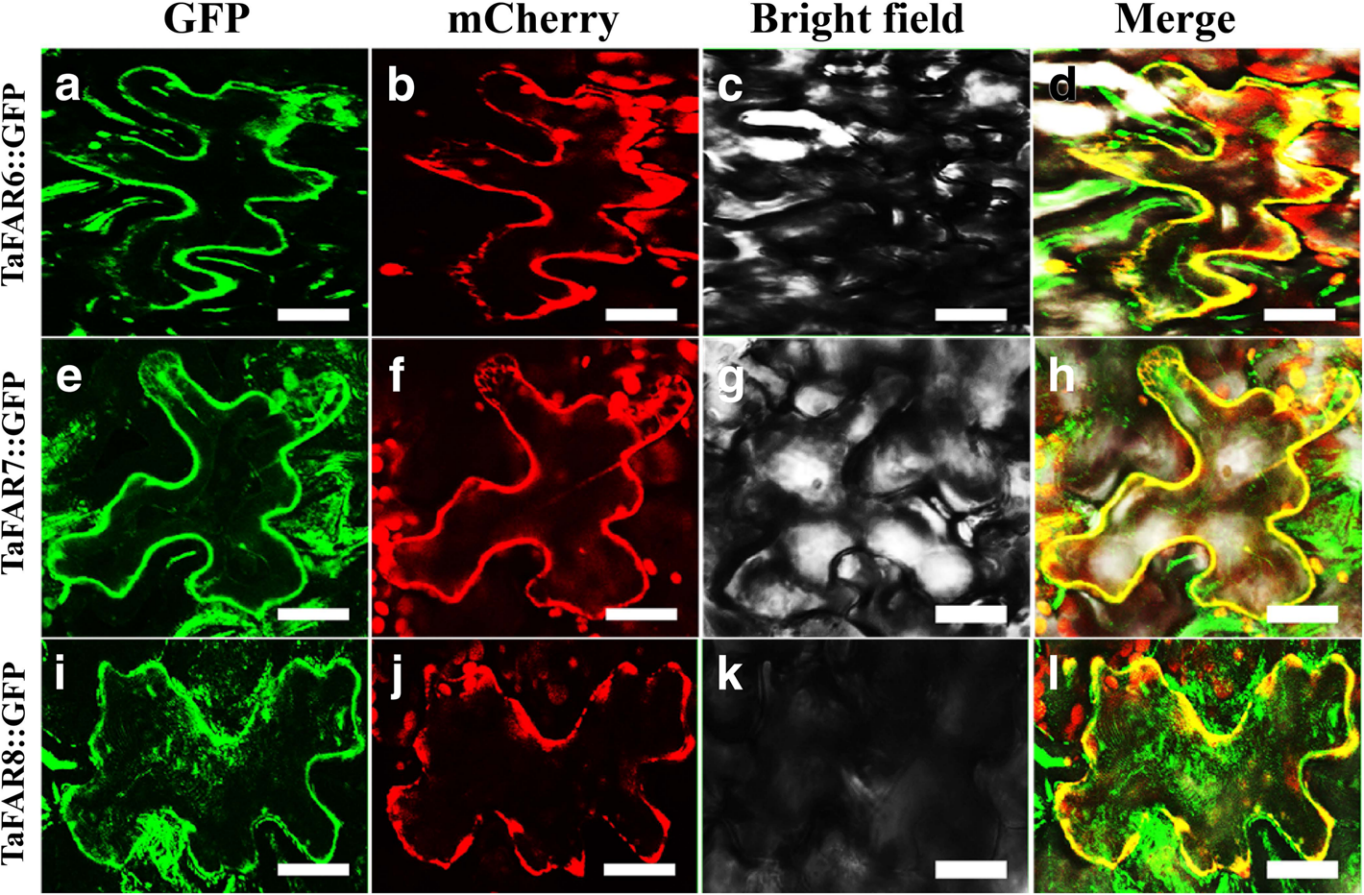
Subcellular localization of TaFAR6, TaFAR7 and TaFAR8 in *Nicotiana benthamiana* epidermal cells. **a**, **e**, **i** GFP fluorescence image of the tobacco epidermal cells expressing 35S:TaFAR-GFP. **b**, **f**, **j** Endoplasmic reticulum (ER) marker mCherry-HDEL is indicated in red. **c**, **g**, **k** Bright-field image of the tobacco epidermal cells. **d**, **h**, **l** Merged image. Bars = 10 μm

Because of the proteins that participate in the biosynthesis of cuticular wax are mainly located in the ER in plant epidermal cells [17, 49]. We predicted that the three TaFARs should be localized to the ER. To confirm this prediction, we generated three constructs harboring the full length opening reading frame without termination codons of TaFARs fused upstream of the green fluorescent protein (GFP) gene under the control of CaMV 35S promoter. In addition, a red fluorescent protein (RFP) mCherry-HDEL was used as an ER indicator [50, 51]. These vectors were co-transformed into *Nicotiana benthamiana* leaf epidermal cells. Confocal microscopic observation showed that the green fluorescent signals of TaFARs were co-localized with the red fluorescent signals of ER-RFP (Fig. 7), suggesting that three TaFARs are ER-localized proteins.

## Discussion

Wheat is one of the important staple crops worldwide. In China, wheat is grown on approximately 24 million hectares with a total annual production of 115 million tons and an average yield of 4.75 tons ha^− 1^ [52, 53]. Wheat is very crucial for providing humans with energy. However, wheat growth is severely affected by biotic and abiotic stresses such as drought and heat. One response of the plant to drought and heat stress is to secrete wax to the epidermal layer [54]. Wax serves as a waterproof barrier and restricts the nonstomatal water loss [18, 55].

Biosynthesis of cuticular wax is a complex biological process that consists of two main stages: the elongation of saturated C_16_ and C_18_ fatty acyl-CoAs and the synthesis of very long-chain fatty acid, aldehydes, alcohols, alkanes, and esters. Although many wax mutants have be identified in arabidopisis [56, 57], the underlying molecular mechanism of wax biosynthesis in wheat is poorly understood. In present studies, we first determined the cuticular wax of wheat leaf blade at the seedling stage. Primary alcohols are the major components, and the C_28_ primary alcohol is the most abundant alcohol. These results are consistent with previously reported findings [21, 41]. Based on the micromorphology of wheat leaf blade at the seedling stage, platelet-shaped wax crystals are attached to epidermal cell, and there is no difference between adaxial and abaxial sides. It is very likely that the lobed plate crystals of cuticular wax are associated with primary alcohols. Similar points were proposed by Gulz and Carver [58, 59].

In plants, numerous genes have been characterized that are involved in biosynthesis of wax metabolism [60, 61]. What genes are responsible for accumulation of long-chain fatty alcohols in wheat? In this study, we identified and cloned three *TaFARs* from wheat leaf blade at the seedling stage, named *TaFAR6*, *TaFAR7* and *TaFAR8*. All of these genes encode a FAR with the predicted NADB domain and sterile domain. Thus, these three *TaFARs* likely exhibit a similar function in catalyzing primary alcohols biosynthesis. Previous studies have indicated that heterogeneous expression of *CER4* in yeast results in the production of C24:0-OH and C26:0-OH [22]. Moreover, AtFAR1, AtFAR4 and AtFAR5 form alcohols with chain lengths ranging from C18:0 to C24:0 in yeast [26]. Expression of *E.gracilis* EgFAR in yeast led to the accumulation of C14:0 and C16:0 primary alcohols [42]. In hexaploid wheat, *TaFAR1*, *TaFAR5*, *TaFAR2*, *TaFAR3* and *TaFAR4* are induced to produce C22:0 or C24:0 to C28:0 primary alcohols [30, 31, 33]. To directly assess the reductase activities of the TaFARs and to investigate their substrate specificities, we expressed the coding regions of three *TaFARs* in yeast. All *TaFAR*-expressing yeast accumulated free very-long-chain fatty alcohols of the characteristic C_24_ and C_26_ length. However, the three *TaFARs* expressed in yeast exhibited differences. The substrate specificity of TaFAR8 was particularly stringent for C_24_ acyl chains. The remaining two *TaFARs* accept a couple of chain lengths and mostly generate C24:0 and C26:0 primary alcohols. These results indicated that *TaFAR6*, *TaFAR7* and *TaFAR8* have a strong preference for the C24:0 and C26:0 very-long-chain fatty acids. These *TaFARs* seemingly could only accept C24:0 and C26:0 fatty acyl-CoAs as substrate in yeast. This phenomenon is very similar to that of *LAG1*, *TaFAR5* and *AtFAR1* [26, 31, 62].

We then performed two experiments in higher plants to further confirm the role of *TaFAR6*, *TaFAR7* and *TaFAR8* in primary alcohol biosynthesis. Transgenic expression of *TaFAR6*, *TaFAR7* and *TaFAR8* led to the accumulation of C_24_ to C_32_ primary alcohols in fruit cuticular wax of tomato and the production of C_24_ to C_30_ primary alcohols in flag leaf wax of rice. These results suggest that *TaFARs* may play a critical role in the biosynthesis of cuticular wax. According to the above results, it is likely that widely varying product profiles in different expression systems may be due to different cellular micro-environments between yeast and plants. Previous studies have also shown that product profiles depend largely on heterologous hosts, and thus substrate availability. For example, C_16_ and C_18_ primary alcohols were produced upon expression of jojoba FAR in *E. coli*, but C_22_ primary alcohol was detected in the seeds of high erucic acid oilseed rape (*Brassica napus*) plants [23]. TAA1a produced saturated C_16_ and mono-unsaturated C_18_ alcohols when expressed in *E. coli*, but saturated C_24_ and C_26_ and mono-unsaturated C_18_ alcohols in transgenic tobacco instead [32]. Expression of CER4 in yeast resulted in the accumulation of C24:0 and C26:0 primary alcohols, but expression of CER4 in *cer4–1* produced C_24_, C_26_, C_28_ and C_30_ primary alcohols [22]. Indeed, in Arabidopsis, *AtFAR3*/*Cer4* [22] and *AtFAR5* [26] have a strict chain length specificity and only accept a few substrates. In contrast, in wheat, six FARs (*TaFAR1*, *TaFAR5*, *TAA1a*, *TaFAR2*, *TaFAR3* and *TaFAR4*) seemingly accept a wide range of substrates depending largely on heterologous hosts used to characterize them [30–33]. We next detect the epidermal wax by SEM. Fortunately, when *TaFARs* were overexpressed in higher plant, tomato and rice, waxes with increased plates were detected on the surface of fruits and leaves. These plate crystals were highly similar to the cuticular wax crystal on wheat leaf blade at the seedling stage. This finding indicates that the three *TaFAR* genes are expressed in transgenic lines. Previous studies demonstrated that plant cuticular wax plays various roles in harsh growth environments [63–65]. One important feature of waxes was to prevent nonstomatal water loss, which is crucial for plant survival under stress conditions. Thus, in the future, we will perform the stress experiments to confirm whether our transgenic lines could adapt to the adverse environmental abiotic factors. We also want to cultivate more drought tolerant wheat varieties via genetic engineering.

In Arabidopsis, the FAE complex generates wax synthetic precursors, and FAR enzymes produce fatty alcohols in the acyl-reduction pathway. In addition, the CER1 and MAH1 enzymes in the decarbonylation pathway [6]. Previously reported wax biosynthetic enzymes, such as OsWSL3 in rice [66], TaFAR2 in wheat [33], ZmGL8 in maize [67], BrWAX1 in cabbage [68], LeCER6 in tomato [69]*,* as well as CsWAX1 and CsWAX2 in cucumber [70, 71], are located in ER membranes. Thus, we anticipated that if these three TaFARs were involved in wax synthesis, these proteins would localize to the ER. Visualization of the functional TaFARs–GFP fusion protein in tobacco leaf epidermal cells revealed that the wheat TaFAR6, TaFAR7 and TaFAR8 are also localized to the ER. Accordingly, it is hypothesized that the ER is the subcellular compartment in which most of the cuticular wax components are deposited [57].

Numerous studies have indicated that various genes are associated with the complex regulatory network of cuticular wax biosynthesis. Most genes, Such as *CER1* and *CER6* in Arabidopsis [72, 73], *OsGL1* in rice [74], and *TaFAR5* in wheat [31], respond to water deficit, sodium chloride, cold and ABA treatments [45, 46, 75]. In our study, all six tested stimuli, including drought, heat, cold, ABA, MeJA and pathogenic fungus, induced the expression of our three *TaFARs* at the transcriptional level (Fig. 4), and these results were consistent with previous studies [31, 76].

In conclusion, our study presents powerful evidence that the three TaFARs encode ER-localized FARs and participate in the production of primary alcohols in cuticular wax biosynthesis of hexaploid wheat. Furthermore, *TaFARs* were associated with responses to drought, cold, heat stresses and powdery mildew infection meanwhile in MeJA and ABA-dependent manner in wheat leaves. *TaFAR6*, *TaFAR7* and *TaFAR8* are very promising genes for future genetic engineering studies aimed at generating cultivars exhibiting stress tolerance.

## Conclusions

The aim of this study was to explore more functional genes involved in the biosynthesis of primary alcohols in hexaploid wheat. Overall, our results showed that *TaFAR6*, *TaFAR7* and *TaFAR8* were involved in the production of very-long-chain primary alcohols in yeast and plants, and were also associated with responses to drought, cold, heat stresses and powdery mildew infection meanwhile in MeJA and ABA-dependent manner in wheat leaves.

## Additional files

Additional file 1: **Table S1.** Primers for vector construction and expression analysis. (XLSX 11 kb)

Additional file 2: **Figure S2.** SDS-PAGE of TaFAR6, TaFAR7 and TaFAR8 in *E. coli*. Arrows indicate the His-TaFAR fusion proteins. The empty vector pET28a is as control. M, protein marker. (PDF 5508 kb)

Additional file 3: **Figure S3.** Mass spectra of primary fatty alcohols. (a, c) The mass spectra from the products of *TaFAR6*, *TaFAR7* and *TaFAR8*. (b)Authentic standard of C24:0-OH. (d) Authentic standard of C26:0-OH. (PDF 1039 kb)

Additional file 4: **Figure S4.** Genetic transformation of *TaFAR6*, *TaFAR7* and *TaFAR8* in tomato cv MicroTom and rice cv Zhonghua 11. a, Schematic representation of constructs used in the transformation experiments. LB, T-DNA left border; 35S polyA, CaMV 35S polyA; Hygromycin, Hygromycin resistance gene; Tnos, NOS terminator; RB, T-DNA right border. b, Plant architecture of T1 transgenic lines at the flowering stage. c, PCR screening of transgenic T1 generation tomato plants by detecting the presence of *TaFARs* genes. d, Expression analysis of three *TaFARs* in different overexpression transgenic lines and CK by qRT-PCR. e, Plant architecture of T1 transgenic rice lines at the filling stage. f, PCR screening of transgenic T1 generation rice plants by detecting the presence of *TaFARs* genes. g, Expression analysis of three *TaFARs* in different overexpression transgenic rice lines and CK by qRT-PCR. (PDF 10567 kb)

Additional file 5: **Figure S5.** Epicuticular wax crystal patterns on fruits and leaves of transgenic tomato detected by SEM. The epicuticular wax crystal patterns on the fruits surfaces (a–d). CK (a), *TaFAR6* overexpression plants (b), *TaFAR7* overexpression plants (c) and *TaFAR8* overexpression plants (d), respectively. The epicuticular wax crystal patterns on the leaves of adaxial surfaces (e–h) and abaxial surfaces (i–l). CK (e, i), *TaFAR6* overexpression plants (f, j), *TaFAR7* overexpression plants (g, k), *TaFAR8* overexpression plants (h, l). CK is the empty pCXSN vector control. Scale bars = 2 μm. (PDF 6000 kb)

Additional file 6: **Figure S6.** Epicuticular wax crystal patterns on flag leaves and sheath of transgenic rice detected by SEM. The epicuticular wax crystal patterns on the leaves of adaxial surfaces (a–d), the leaves of abaxial surfaces (e–h) and the sheath surfaces (i–l). CK plants (a, e, i), *TaFAR6* overexpression plants (b, f, j), *TaFAR7* overexpression plants (c, g, k), *TaFAR8* overexpression plants (d, h, j). CK is the empty pCXSN vector control. Scale bars = 2 μm. (PDF 9005 kb)

## Abbreviations

ABA: Abscisic acid
FAR: Fatty acyl-coenzyme A reductase
GC-MS: Gas chromatography-mass spectrometry
kD: kilo dalton
MeJA: Jasmonic Acid Methyl ester
SEM: Scanning electron microscopy
