# Supplementary figures and images for: Three endoplasmic reticulum-associated fatty acyl-coenzyme a reductases were involved in the production of primary alcohols in hexaploid wheat (Triticum aestivum L.)
Source: BMC Plant Biol. 2018 Mar 5;18:41. doi: 10.1186/s12870-018-1256-y (PMC5836450; doi:10.1186/s12870-018-1256-y)

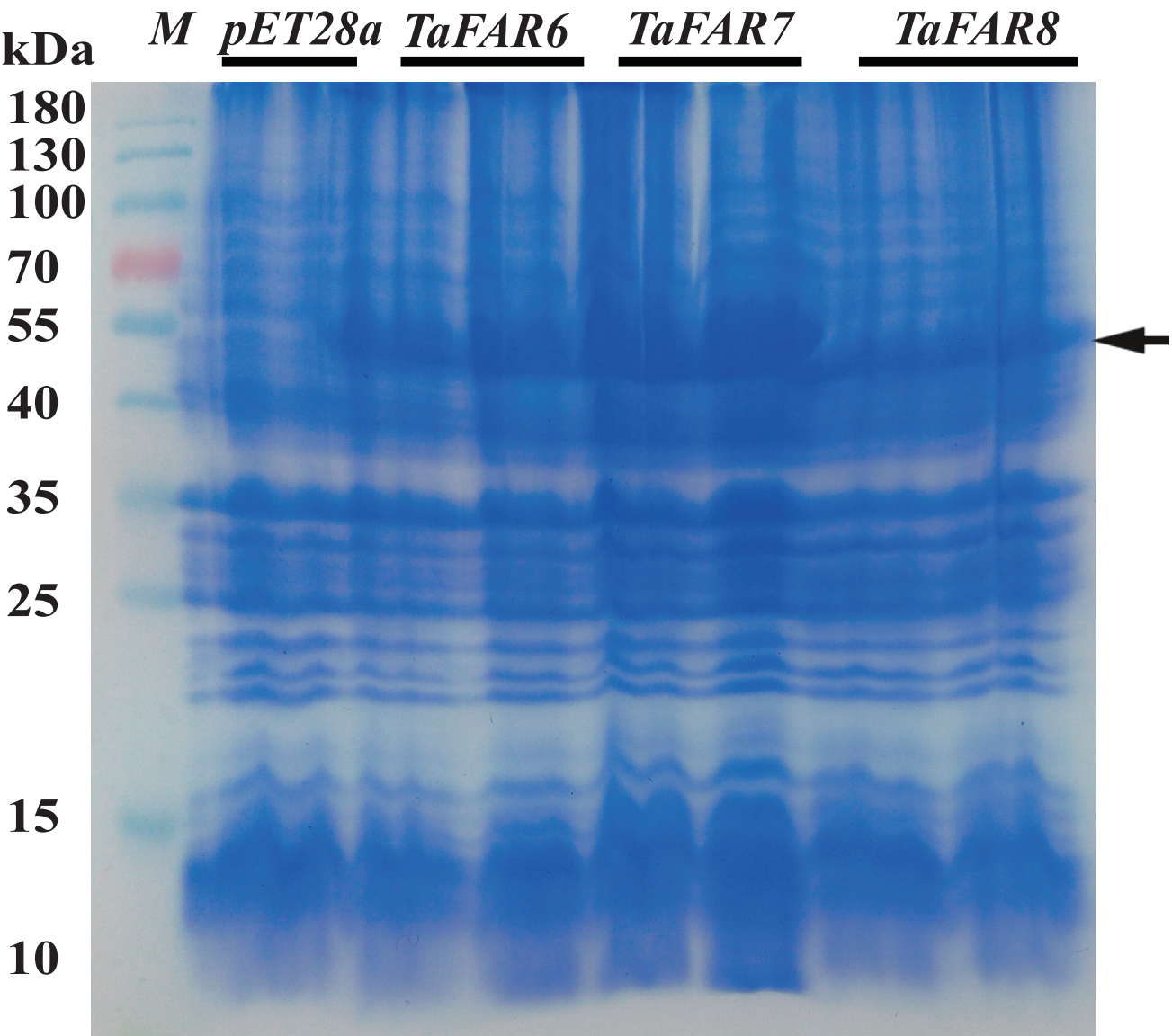

Supplement: Supplementary file 2 — Figure S2. SDS-PAGE of TaFAR6, TaFAR7 and TaFAR8 in E. coli. Arrows indicate the His-TaFAR fusion proteins. The empty vector pET28a is as control. M, protein marker. (PDF 5508 kb) [file 12870_2018_1256_MOESM2_ESM.pdf]

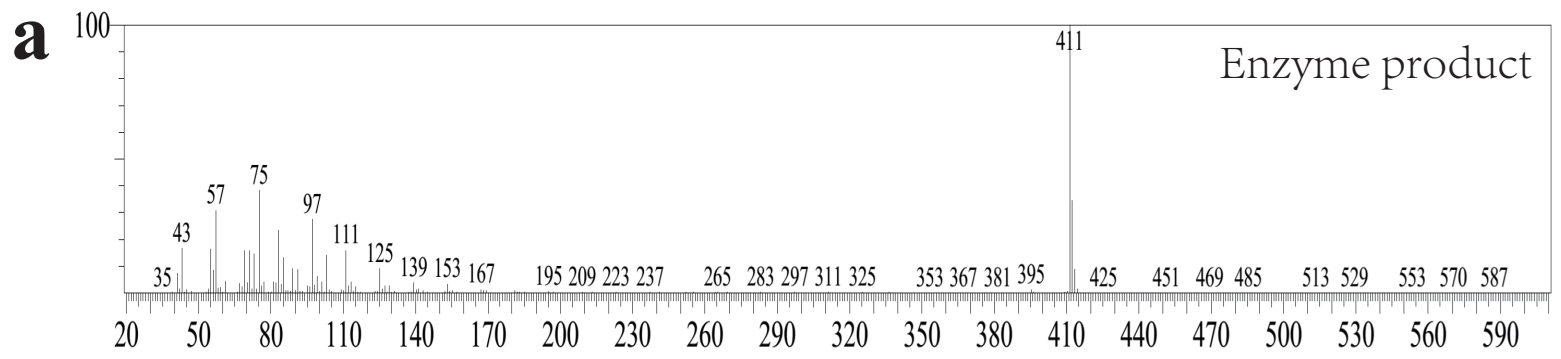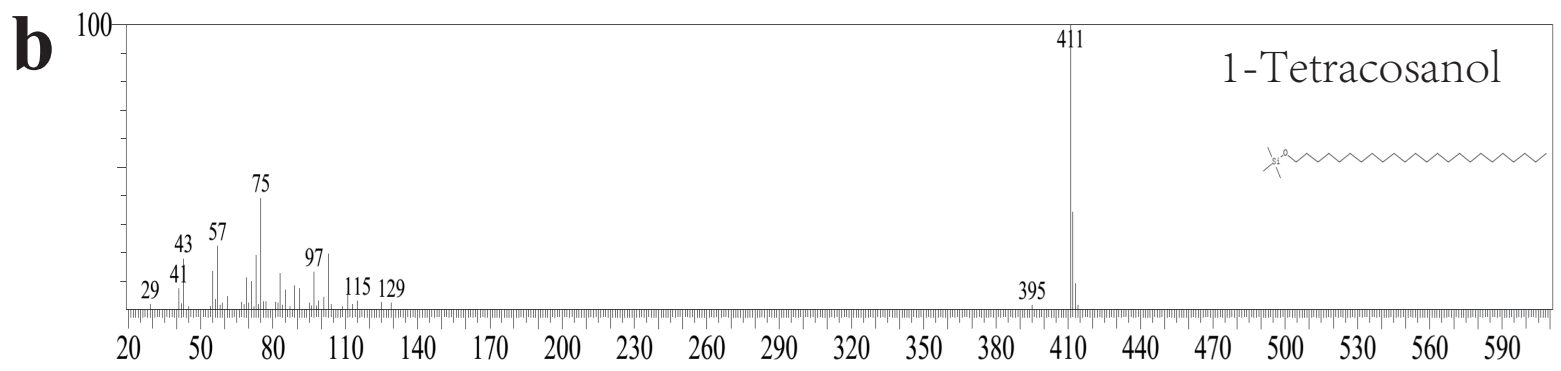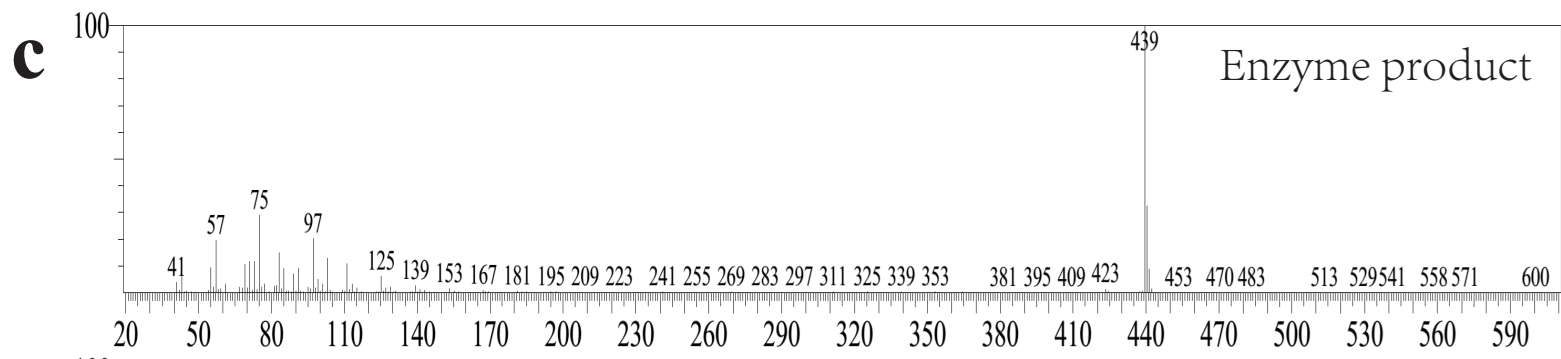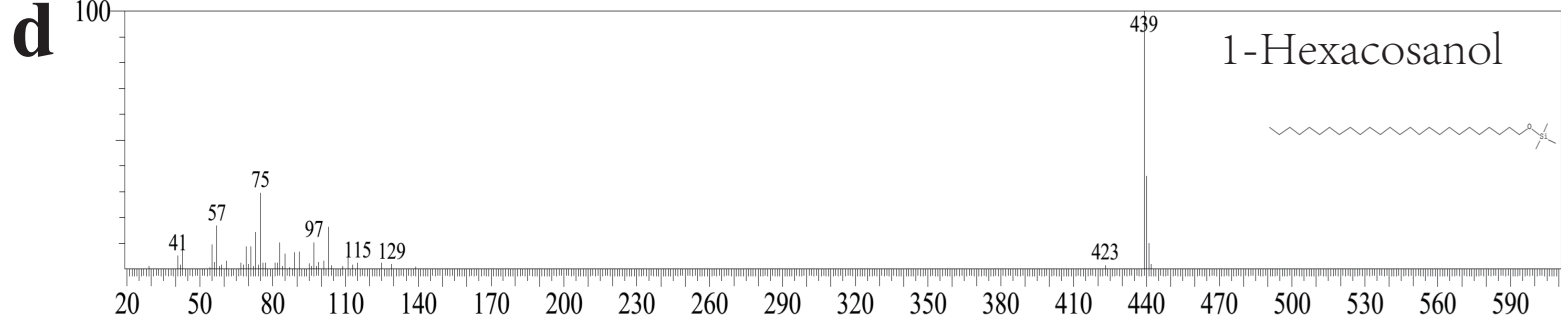

Supplement: Supplementary file 3 — Figure S3. Mass spectra of primary fatty alcohols. (a, c) The mass spectra from the products of TaFAR6, TaFAR7 and TaFAR8. (b)Authentic standard of C24:0-OH. (d) Authentic standard of C26:0-OH. (PDF 1039 kb) [file 12870_2018_1256_MOESM3_ESM.pdf]

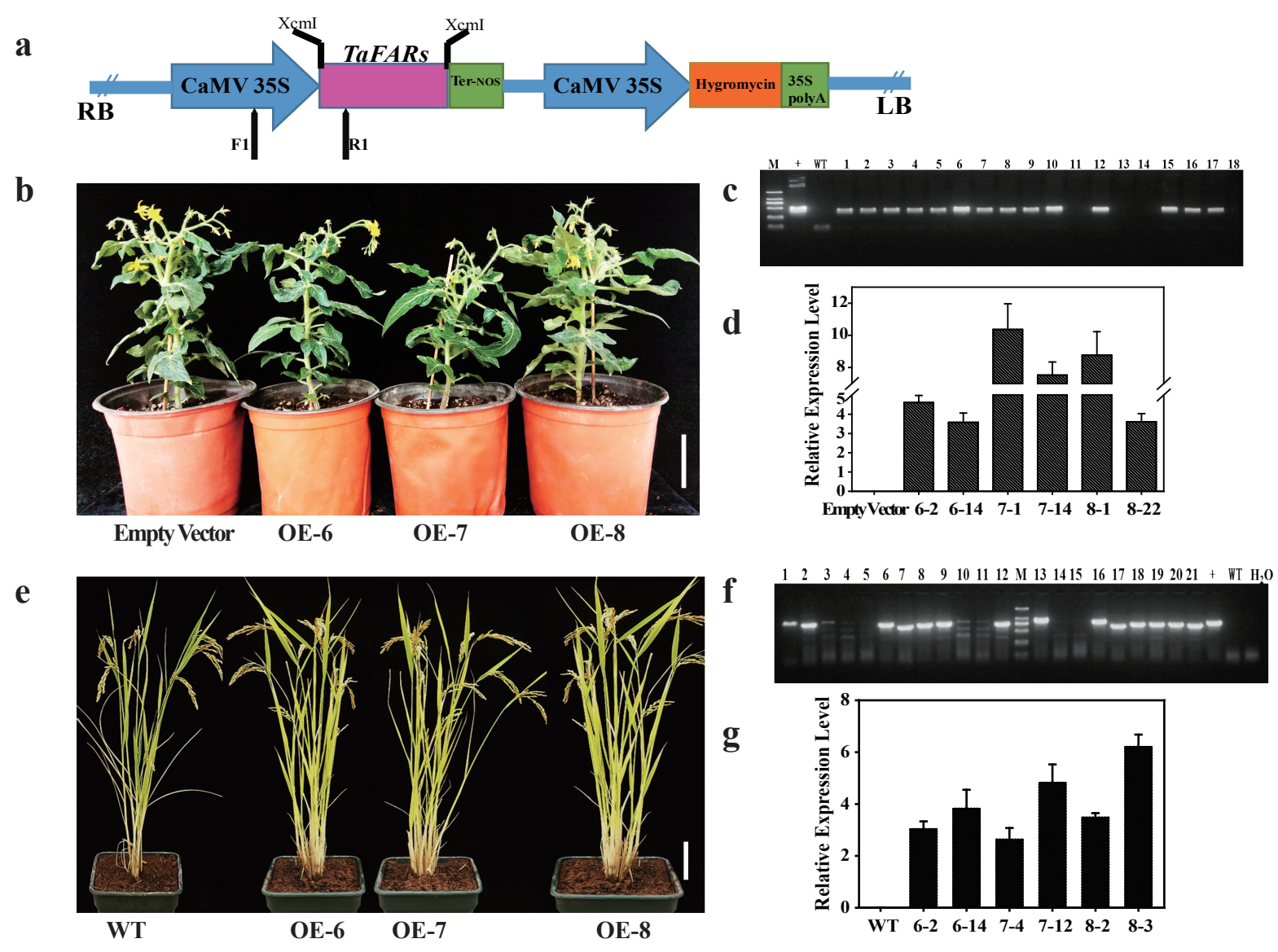

Supplement: Supplementary file 4 — Figure S4. Genetic transformation of TaFAR6, TaFAR7 and TaFAR8 in tomato cv MicroTom and rice cv Zhonghua 11. a, Schematic representation of constructs used in the transformation experiments. LB, T-DNA left border; 35S polyA, CaMV 35S polyA; Hygromycin, Hygromycin resistance gene; Tnos, NOS terminator; RB, T-DNA right border. b, Plant architecture of T1 transgenic lines at the flowering stage. c, PCR screening of transgenic T1 generation tomato plants by detecting the presence of TaFARs genes. d, Expression analysis of three TaFARs in different overexpression transgenic lines and CK by qRT-PCR. e, Plant architecture of T1 transgenic rice lines at the filling stage. f, PCR screening of transgenic T1 generation rice plants by detecting the presence of TaFARs genes. g, Expression analysis of three TaFARs in different overexpression transgenic rice lines and CK by qRT-PCR. (PDF 10567 kb) [file 12870_2018_1256_MOESM4_ESM.pdf]

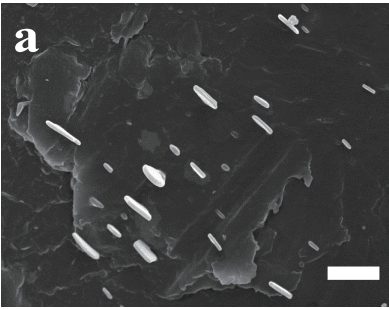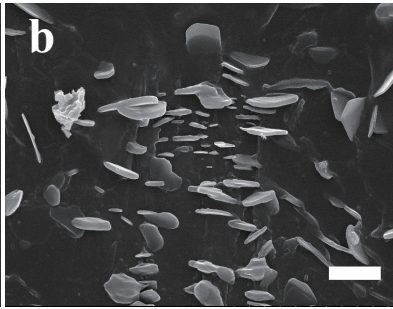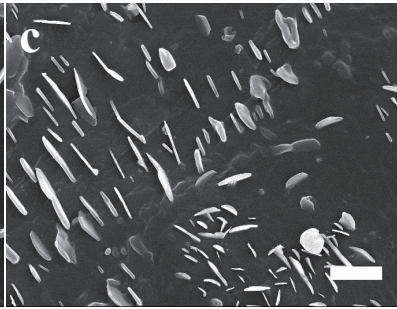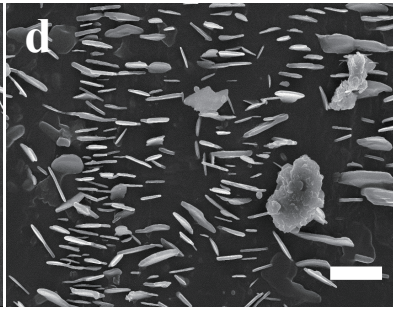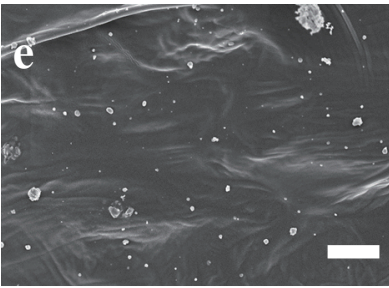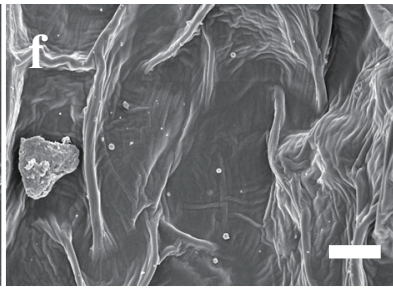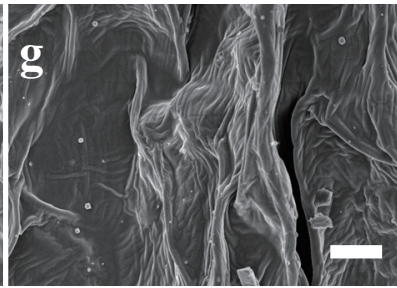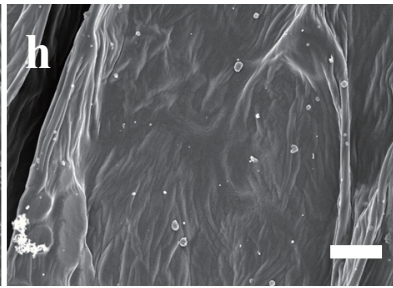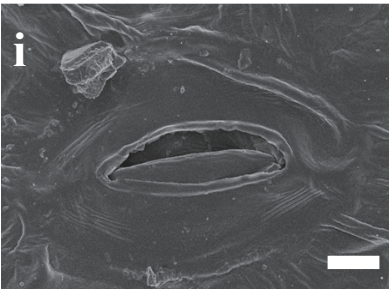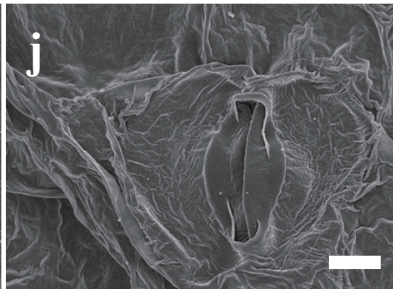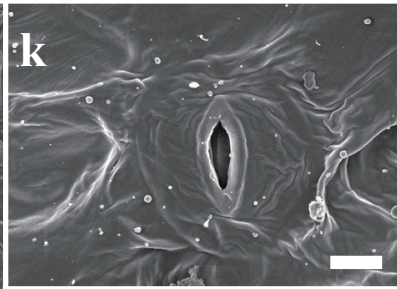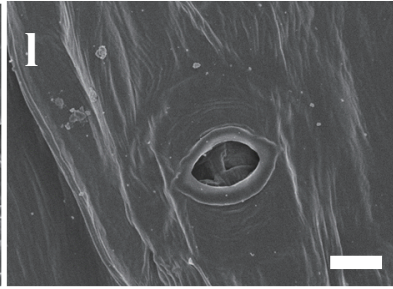

Supplement: Supplementary file 5 — Figure S5. Epicuticular wax crystal patterns on fruits and leaves of transgenic tomato detected by SEM. The epicuticular wax crystal patterns on the fruits surfaces (a–d). CK (a), TaFAR6 overexpression plants (b), TaFAR7 overexpression plants (c) and TaFAR8 overexpression plants (d), respectively. The epicuticular wax crystal patterns on the leaves of adaxial surfaces (e–h) and abaxial surfaces (i–l). CK (e, i), TaFAR6 overexpression plants (f, j), TaFAR7 overexpression plants (g, k), TaFAR8 overexpression plants (h, l). CK is the empty pCXSN vector control. Scale bars = 2 μm. (PDF 6000 kb) [file 12870_2018_1256_MOESM5_ESM.pdf]

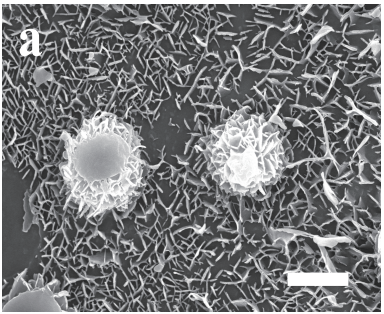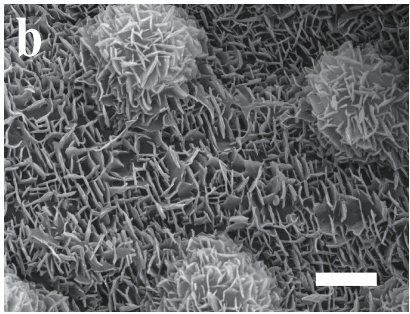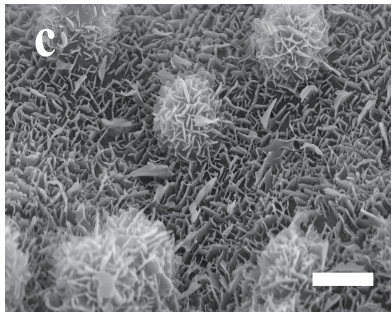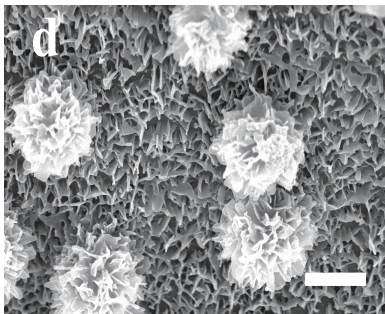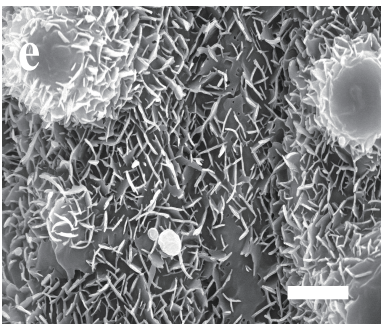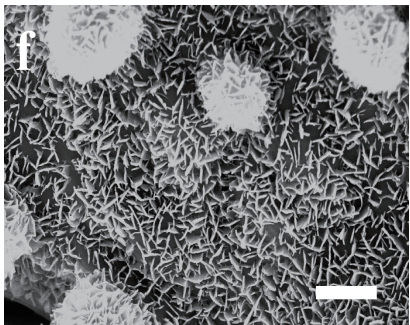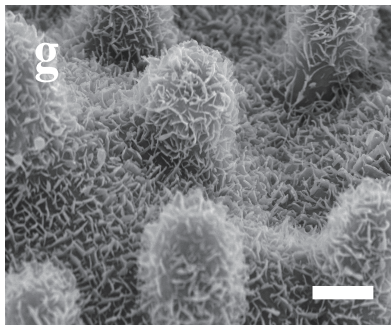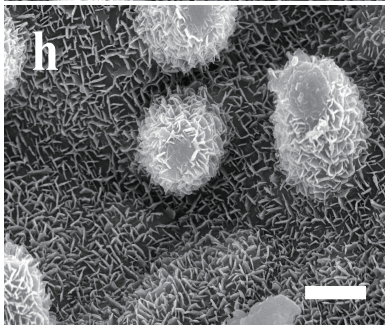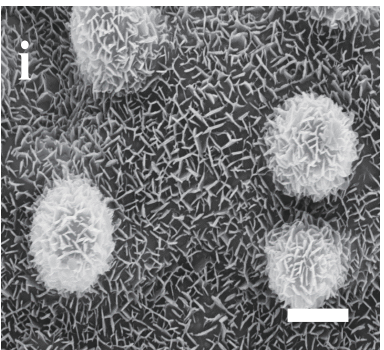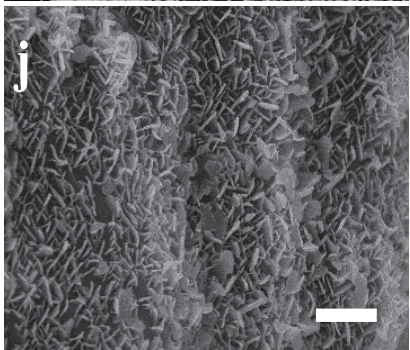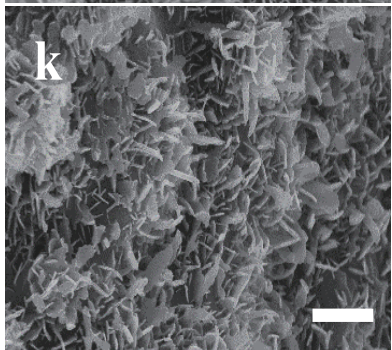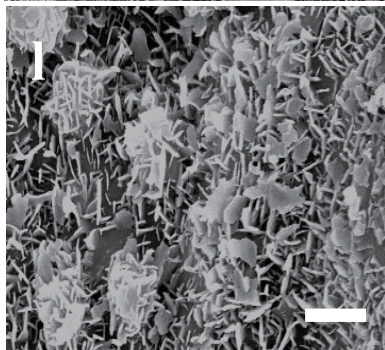

Supplement: Supplementary file 6 — Figure S6. Epicuticular wax crystal patterns on flag leaves and sheath of transgenic rice detected by SEM. The epicuticular wax crystal patterns on the leaves of adaxial surfaces (a–d), the leaves of abaxial surfaces (e–h) and the sheath surfaces (i–l). CK plants (a, e, i), TaFAR6 overexpression plants (b, f, j), TaFAR7 overexpression plants (c, g, k), TaFAR8 overexpression plants (d, h, j). CK is the empty pCXSN vector control. Scale bars = 2 μm. (PDF 9005 kb) [file 12870_2018_1256_MOESM6_ESM.pdf]
